# Alpha lipoic acid attenuates evoked and spontaneous pain following surgical skin incision in rats

**DOI:** 10.1080/19336950.2021.1907058

**Published:** 2021-04-12

**Authors:** Sonja Lj. Joksimovic, Nathan Lamborn, Vesna Jevtovic-Todorovic, Slobodan M. Todorovic

**Affiliations:** a Department of Anesthesiology, University of Colorado Denver Anschutz Medical Campus, Aurora, CO, USA; b Neuroscience Graduate Program, Graduate Program in Pharmacology, and Graduate Program in Biomedical Sciences, University of Colorado Denver, Anschutz Medical Campus and Rocky Mountain Regional VA Medical Center, Aurora, CO, USA

**Keywords:** Postsurgical pain, T-type calcium channel, acute pain, Alpha lipoic acid, novel analgesics

## Abstract

Our previous studies have implicated Ca_V_3.2 isoform of T-type Ca2+ channels (T-channels) in the development of postsurgical pain. We have also previously established that different T-channel antagonists can alleviate in vivo postsurgical pain. Here we investigated the analgesic potential of another T-channel blocker and endogenous antioxidant molecule, α-lipoic acid (ALA), in a postsurgical pain model in rats. Our in vivo results suggest that single and repetitive intraperitoneal injections of ALA after surgery or preemptively, significantly reduced evoked mechanical hyperalgesia following surgical paw incision. Furthermore, repeated preemptive systemic injections of ALA effectively alleviated spontaneous postsurgical pain as determined by dynamic weight-bearing testing. We expect that our preclinical study may lead to further investigation of analgesic properties and mechanisms of analgesic action of ALA in patients undergoing surgery.

## Introduction

Most commonly used therapeutics for alleviation of intraoperative as well as acute postsurgical pain are local anesthetics, nonsteroidal anti-inflammatory drugs (e.g. inhibitors of cyclo-oxygenase) and opioids. However, despite the fact that these powerful therapeutics alleviate acute postoperative pain, 10–50% of the patients experience persistent pain after surgery [1]. This condition represents a vastly unrecognized clinical problem, and if not treated, can progress to chronic postsurgical pain, lasting even 3–6 months after the surgery. Furthermore, chronic use of conventional analgesics to alleviate persistent postsurgical pain is not devoid of serious adverse events [2]. In 20% of the patients undergoing surgery, opioids are well known to induce their adverse effects, such as gastrointestinal effects, central nervous system effects, pruritus, or urinary retention not to mention tolerance and addiction. Therefore, there is an ongoing recognized need to implement measures to reduce the use of opioids after surgery, which will decrease the incidence of adverse events, and also consequently, reduce treatment costs associated with surgery and improve patient care. There is also a clear need for the development of novel therapeutics for treating intractable pain with greater efficacy and with lesser adverse events.

Alpha lipoic acid or ALA (1,2-dithiolane-3-pentanoic acid, 1,2-dithiolane-3-valeric acid, 5-(1,2-dithiolan-3-yl) valeric acid, 6,8-thioctic acid) naturally occurs in living organisms as a cofactor for many mitochondrial enzymes involved in energy metabolism. Both in vivo and in vitro studies have shown that ALA is important for various intracellular processes such as scavenging free radicals, chelating redox-active transition metals, regulating the detoxification of heavy metals and modulating various signal transduction pathways in physiological and pathological conditions [3]. ALA exists as two enantiomers (R and S) due to the presence of a chiral center at the C6 location; however only R-ALA can be endogenously found. In humans, R-ALA is an essential cofactor for several mitochondrial enzyme complexes necessary for energy production and the catabolism of alpha-keto acids and amino acids [4,5].

Importantly, ALA has been recognized as a therapeutic option for patients with diabetic neuropathy. It has been shown that chronic application of ALA during a period of 6 months in patients suffering from diabetic neuropathy is effective, safe, and well tolerated [6]. Furthermore, ALA has also been shown to reduce pain in patients suffering from chemotherapy-induced peripheral neuropathy [7]. Several studies have also shown an analgesic potential of ALA in preclinical neuropathy pain models in rodents, such as diabetic neuropathy [8,9] and paclitaxel induced neuropathy [10]. The exact mechanism of analgesic efficacy of ALA is not yet fully understood but it is likely that multiple mechanisms may be involved. In paclitaxel induced neuropathy in rodents, ALA reduced neuropathy and oxidative stress by targeting the Nrf2 pathway [10]. In streptozotocin-induced pain model in rats, ALA in combination with coenzyme Q10 suppressed diabetic neuropathy – induced oxidative stress by decreasing lipid peroxidation and reactive oxygen species, and increasing glutathione in DRG neurons [11]. In a mouse model of multiple sclerosis-induced pain, it was proposed that ALA exerted antiallodynic effect by attenuating upregulated BDNF-TrkB-ERK signaling in the dorsal horn of the spinal cord [12].

The role of low-voltage-activated calcium channels or T-type channels (T-channels) in pain is well established [13,14]. T-channels are implicated in the development of diabetic neuropathy [15–19], as well as in an acute postsurgical pain model [20]. Previous studies have shown that the activity of T-channels has been upregulated in dorsal root ganglion neurons in experimental models of nerve injury [15,21,22], diabetic neuropathy [23] and visceral pain [24]. We identified the Ca_V_3.2 isoform of T-channels to be an important contributor to hyperexcitability of cell bodies of nociceptive peripheral sensory neurons in incised rats. Furthermore, in vivo experiments revealed that selective T-channel blockers, such as TTA-P2 reduced thermal and mechanical hyperalgesia after surgical incision in rats [20].

In an effort to pursue endogenous molecules with T-channel blocking properties, our group previously discovered that ALA exerts its in vivo antinociceptive properties by inhibiting Ca_V_3.2 T-channels in peripheral nociceptors and consequently by reducing T-channel-dependent cellular excitability in acutely isolated rat sensory neurons [25]. Here we sought to investigate the potential of ALA to treat acute evoked and spontaneous pain in rats after surgical skin incision of the hind paw.

## Materials and methods

### Animals

Experimental protocols were approved by the Animal Care and Use Committee of the University of Colorado Anschutz Medical Campus, and all experiments were done in accordance with the Guide for the Care and Use of Laboratory Animals (Institute of Laboratory Animal Resources, 1996). All animals were housed 2 per cage, on a 12 h light-dark cycle with food and water ad libitum. Female Sprague-Dawley (Envigo, Indianapolis, IN, USA) rats weighing 200–240 grams were used in our studies.

On each experimental day, animals were randomly assigned to treatment groups with the experimenter blinded to drug treatment. All efforts were made to reduce animal suffering and the number of animals used. Whenever possible, studies were designed to generate groups of equal size, using randomization and blinded analysis [20,26].

### Drugs

Either a racemic mixture (Sigma-Aldrich, St. Louis, MO) or the R enantiomer of ALA with improved water solubility (GeroNova Research Inc, Fairfax, CA) were used in our experiments. ALA was dissolved in saline (BaxterHealthcare Corporation, Deerfield IL, USA) and administered systemically via intraperitoneal (i.p.) injection. We have chosen doses of 60 and 120 mg/kg that were shown in a recent study to be effective in suppressing visceral pain responses in rats [9].

For single-drug injections, we performed baseline measurements of mechanical sensitivity before injections, and then at the time points of 30, 60, and 90 minutes postinjections. For all experiments with repeated injections pain testing was done at least 24 hours after the last injection of ALA up to seven 7 days for mechanical hyperalgesia and at POD 1 for spontaneous pain assessment.

### Incisional pain model

To study analgesic properties of ALA in an acute somatic pain model, we used the incisional pain model in rats that has been described previously [27]. In brief, animals were anesthetized with isoflurane (2 to 3%), and the plantar surface of the right paw was incised longitudinally. The underlying plantaris muscle was elevated and incised, after which the skin was closed with two sutures. Each animal was allowed to recover individually in a cage, and all experiments were initiated as early as 2 hours after incision. Full details are provided in our recent publications [19,26].

### Mechanical hyperalgesia

In order to determine the development of mechanical hyperalgesia in rats, we used the electronic Von Frey apparatus (Ugo Basile, Varese, Italy). The apparatus utilizes a single rigid filament that exerts pressure to the plantar surface of the paw in a range from 0 to 50 grams. Animals were placed in plastic enclosures on a wire mesh stand to habituate for 15 minutes. After habituation, a probe was applied to the plantar surface of the paw through the mesh floor of the stand, and constant force was applied to the mid-plantar area of the paw. As soon as the exerted pressure of the punctate stimulus reaches the maximum force that the animal can endure, immediate brisk paw withdrawal is noticeable, and the force in grams is displayed on the apparatus representing a threshold for paw withdrawal response (PWR). Each paw was tested three times and the average value of threshold PWRs was used for further analysis. Any other voluntary movement of the animal was not considered as a response.

### Spontaneous pain assessment

Spontaneous pain in rats after surgical paw incision was assessed by measuring the weight bearing of the incised paws with a dynamic weight bearing (DWB) device (Bioseb, Boulogne, France) as described previously [28]. This apparatus consists of a Plexiglas enclosure (22 × 22 × 30 cm) with a floor sensor composed of 44 × 44 captors (10.89 mm^2^ per captor). A camera was attached to an enclosure cover enabling constant monitoring of freely moving rats during a 5 minute trial. The floor sensor collects the pressure data and after 5 minute trial period both data and live recording were transmitted to a laptop computer via a USB interface. Raw pressure and visual data were analyzed with the DWB software. For each time segment where the weight distribution was stable for more than 0.5 s, detected pressure zones were then validated and assigned by blinded experimenter as either right or left hind paw or front paw according to the video. Data are expressed as the mean value of the weight on every limb during the complete testing period. Animals were not acclimatized to the enclosure before the initial testing period in order to maximize exploration behaviors.

### Rotarod for the assessment of coordination

Balance and motor coordination in rats after receiving ALA was assessed using rotarod test for rats (Ugo Basile, Milano, Italy). Rats were placed on a revolving rod rotating at a constant speed of 15 rpm [29,30] and time spent on a rod without falling was measured. A cutoff of 60 s was considered as the maximum time spent on the rod without falling. The animals were tested to establish baseline performance, and then retested 30 minutes after receiving 120 mg/kg i.p. of ALA, which was the highest dose used in our experiments.

### Data analysis and statistics

The data and statistical analysis comply with the recommendations on experimental design and analysis in pharmacology [31,32]. The declared group size is the number of independent values, and statistical analysis was done using these independent values. For each experiment, animals were randomly assigned to experimental groups in order to generate biological replicates, and the experimenter was blinded for the treatment until subsequent data analyses have been performed. No outliers were excluded in these experiments. For all studies animals were litter-matched and age-matched to keep the treatment groups as similar as possible. Each data point from experiments was expressed as mean ± SEM. Proper statistical analysis of the differences in effects between the treatment and the vehicle groups was performed using two-way RM ANOVA followed by Tukey’s, and Bonferroni’s post-hoc tests as appropriate. In multigroup studies with parametric variables, post hoc tests (recommended by GraphPad prism) were conducted only if F in ANOVA achieved the necessary level of statistical significance and there was no significant variance in homogeneity. Signiﬁcant differences between group means are indicated when p < 0.05. GraphPad Prism 7 (GraphPad Software, La Jolla, CA, USA) was used for all statistical analyses.

## Results

### Effects of single systemic application of ALA on postsurgical mechanical hyperalgesia in rats

**Figure 1.**
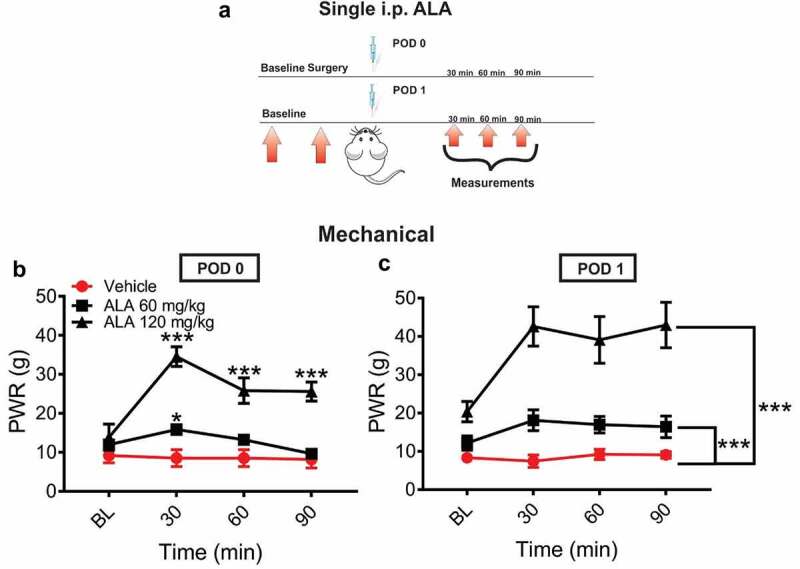
Single systemic ALA reduces mechanical hyperalgesia after incision

In order to investigate the analgesic effect of ALA in postsurgical pain model, rats’ paws were surgically incised and ALA was injected intraperitoneally (i.p.) either two hours (postoperative day 0 POD 0) or 24 hours (postoperative day 1 POD 1) after surgery in two doses (60 mg/kg i.p. and 120 mg/kg i.p.; Figure 1a). After surgical incision, systemic ALA applied as a single dose of 60 mg/kg i.p. at two hours exerted a transient antihyperalgesic effect only at 30 minutes postinjection, while dose of 120 mg/kg i.p. exerted a more impressive and prolonged antihyperalgesic effect over the time course of 90 minutes postinjection (Figure 1b). When ALA was applied on POD 1, both doses exerted a statistically significant and dose-dependent decrease in mechanical hyperalgesia during 90 minutes after treatment application (Figure 1c), when compared to the vehicle group.

A. A time course of experimental protocol for a single application of ALA. B. ALA applied 2 h after surgery on postoperative day (POD) 0 at both doses of 60 mg/kg and 120 mg/kg i.p. induced significant antihyperalgesic effect in a dose-dependent manner as compared to the vehicle group (n = 5–8 rats, two-way RM-ANOVA, interaction: F(4,32) = 4.12; p = 0.0084; treatment: F(2,16) = 38.25; p < 0.001). C. ALA applied 24 h after surgery on POD 1 in both doses of 60 mg/kg and 120 mg/kg i.p. induced significant antihyperalgesic effect in a dose-dependent manner as compared to the vehicle group (n = 5–8 rats, two-way RM-ANOVA, interaction: F(4,34) = 1.82; p = 0.15; treatment: F(2,17) = 31.17; p < 0.001). Data are presented as means ± SEM. *p < 0.05; **p < 0.01; ***p < 0.001.

**Figure 2.**
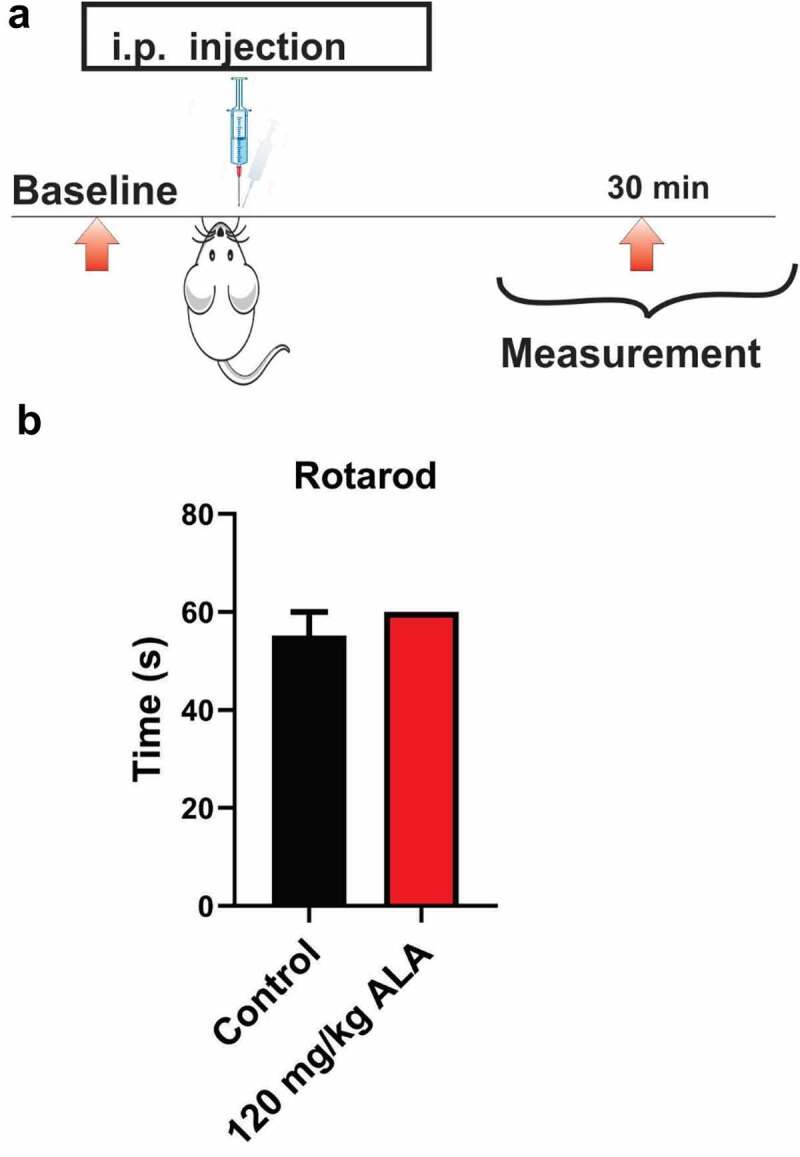
Systemic ALA in a dose of 120 mg/kg i.p does not affect motor performance in rats

We noticed that rats injected with the higher dose of ALA exhibited a transient state resembling sedation. Hence, we next evaluated the potential of the highest tested dose of ALA applied i.p. (120 mg/kg) to impair motor performance of injected rats 30 minutes postinjection (Figure 2a). The results of our experiments revealed that the highest tested dose of ALA in our experiments (120 mg/kg i.p.) did not affect balance and coordination of the rats on the rotarod (n = 4, paired; t-test, p = 0.391) at 15 rpm (Figure 2b).

A. A time course of experimental protocol of application of ALA and rotarod assessment. B. ALA applied i.p. at a dose of 120 mg/kg did not affect the time animals spent on rotarod 30 minutes after injections, as compared to the preinjection baseline conditions (n = 4 rats, p = 0.391, paired t-test). Data are presented as means ± SEM.

### Effects of repeated systemic application of ALA on postsurgical mechanical hyperalgesia in rats

**Figure 3.**
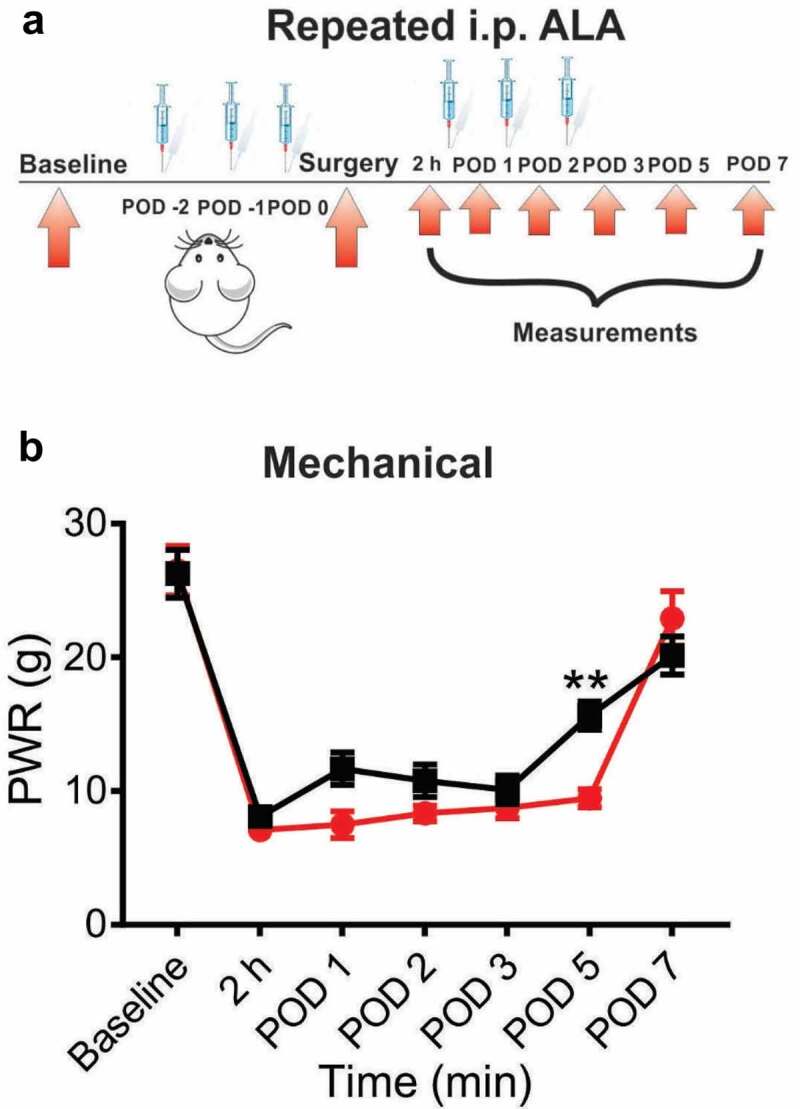
Repeated systemic ALA in a dose of 60 mg/kg had mild effect on mechanical hyperalgesia after incision

In another set of experiments we investigated the antihyperalgesic effect of repeated application of ALA in our established postsurgical pain model. For these experiments, ALA was applied once daily i.p. during three days before the surgery, and on POD 0, POD 1 and POD 2 (postoperative day 2). Repeated systemic application of 60 mg/kg of ALA significantly attenuated mechanical hyperalgesia in rats as compared to the vehicle group (Figure 3a and 3b). However, the antihyperalgesic effect on mechanical hyperalgesia was noticeable only on POD 5.

A. A time course of experimental protocol for a repeated application of ALA. B. ALA applied i.p. in a dose of 60 mg/kg, as three consecutive doses before, and three consecutive doses after surgery, exerted small but significant antihyperalgesic effect in the test of mechanical sensitivity on POD 5, as compared to the vehicle group (n = 5–8 rats, two-way RM-ANOVA, interaction: F(5,55) = 4.01; p = 0.036; treatment: F(1,11) = 5.573; p = 0.048). Data are presented as means ± SEM. *p < 0.05; **p < 0.01; ***p < 0.001.

**Figure 4.**
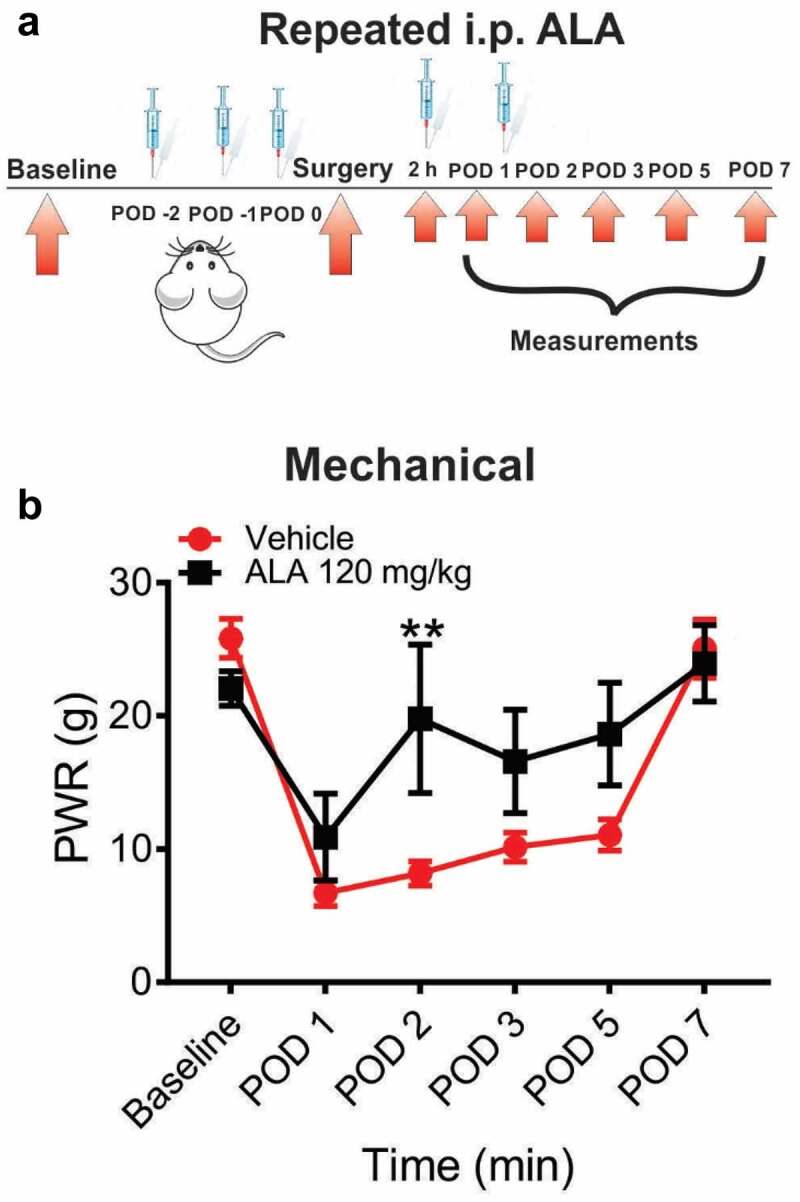
Repeated systemic ALA in a dose of 120 mg/kg i.p. reduces mechanical hyperalgesia after incision

In another set of experiments, we investigated the antihyperalgesic effect of systemic repeated application of 120 mg/kg of ALA. This time, ALA was applied as three consecutive doses before the surgery, and two additional doses on POD 1 and POD 2 (Figure 4a). Our data showed that systemic repeated application of ALA at a dose of 120 mg/kg i.p. led to an increase of baseline paw withdrawal thresholds to a mechanical stimulus almost to the preincision baseline value, indicating that ALA elicited a robust antihyperalgesic effect on mechanical hyperalgesia in rats on POD 2 (Figure 4b).

A. A time course of experimental protocol for a repeated application of ALA. B. ALA applied i.p. at a dose of 120 mg/kg, as three consecutive doses before, and two consecutive doses after surgery, exerted a significant antihyperalgesic effect in the test of thermal sensitivity on POD 2, as compared to the vehicle group (n = 4–7 rats, two-way RM-ANOVA followed by Sidak’s post-hoc test, interaction: F(4,36) = 3.473; p = 0.0169, at POD2 p = 0.0073; treatment: F(1,9) = 4.927; p = 0.0536). Data are presented as means ± SEM. **p < 0.01.

Preemptive applications of ALA effectively abolished spontaneous pain in rats

**Figure 5.**
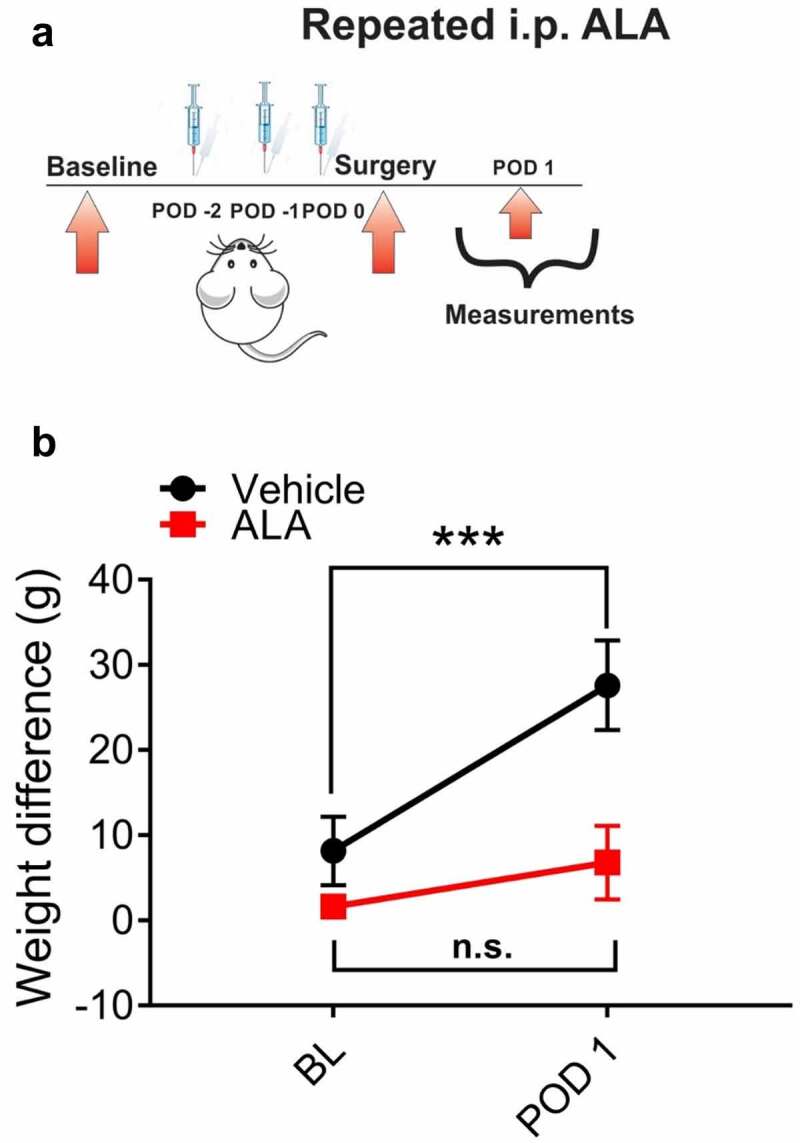
Repeated systemic ALA in a dose of 120 mg/kg i.p. reduces spontaneous pain after incision

In order to investigate if preemptive treatment with ALA affects spontaneous pain in rats after surgical incision, we systemically applied ALA before surgery at three consecutive doses of 120 mg/kg (i.p.) daily, and tested the pressures of the incised and non-incised paws exerted against the floor sensor in the dynamic weight-bearing test chamber (Figure 5a). We discovered that preemptive repeated application of ALA effectively reduced the differences in weight-bearing scores between incised and unincised paws at POD 1, as compared to the vehicle group (Figure 5b).

A. A time course of experimental protocol for a repeated application of ALA. B. ALA applied i.p. at a dose of 120 mg/kg, as three consecutive doses before surgery, reduced the differences in weight bearing between incised and unincised paws at POD 1, as compared to the vehicle group (n = 6–9 rats per group, ***p < 0.001, two-way RM-ANOVA with Sidak’s post-hoc test, Interaction: F(1,13) = 5.157, p = 0.041; exact p value for POD 1 timepoint: p < 0.001; treatment: F(1,13) = 6.2, p = 0.027). Data are presented as means ± SEM.

## Discussion

### ALA is effective analgesic agent alleviating evoked postsurgical pain in rats

Recent animal and human studies have shown that symptoms of stimulus evoked pain (thermal and mechanical hyperalgesia), as well as spontaneous pain symptoms such as guarding are prominent after surgical procedures [33]. Our results presented herein demonstrate that systemic application of ALA can successfully alleviate postsurgical pain in rodents and is devoid of any significant effect on motor performance.

Acute painful episodes from surgical skin incision and deep tissue injury are generally responsive to conventional treatments such as opioid analgesics and regional anesthesia with local anesthetics. However, when administered chronically these treatments can produce serious side effects such as numbness and muscle weakness from local anesthetics as well as respiratory depression, dependence, and addiction from the excessive use of opioids. In addition, it has been well established that long-term use of opioids can lead to a heightened pain sensitivity state termed opioid-induced hyperalgesia, and that can paradoxically increase postoperative pain [34,35]. Thus, further mechanistic studies of sensitization of postoperative pain responses manifested as hyperalgesia, allodynia, and spontaneous pain are warranted.

Here, we specifically focused on punctate mechanical hyperalgesia and spontaneous pain measures, given that they are the most relevant to clinical setting [36–38]. We have previously established that Ca_V_3.2 channels play an important role in the development of both thermal and mechanical hyperalgesia in the incisional pain model in rats and mice [19,20,26]. We also demonstrated that hyperalgesia develops to a lesser extent in Ca_V_3.2 knock-out (KO) mice as compared to WT mice [20], and that T-channel blockers can successfully alleviate both thermal and mechanical hyperalgesia after surgical incision in rats [19,20,26]. Our earlier study [25] demonstrated that ALA reduced T-currents in freshly dissociated cell bodies of sensory neurons in dorsal root ganglia of rats. Furthermore, we were able to show that local intraplantar injection of ALA reduced sensitivity to noxious thermal and mechanical stimuli in wild-type but not in Ca_V_3.2 KO mice indicating that ALA exerts its analgesic properties by modulating the function of T-channels [25]. Our ensuing molecular studies demonstrated that ALA inhibited recombinant Ca_V_3.2 currents in HEK-293 cells by reversibly oxidating specific thiol residues on the cytoplasmic face of the channel. We also demonstrated in this study that unlike many traditional ion channel blockers that typically inhibit T-currents completely such as TTA-P2 (3,5-dichloro-N-[1-(2,2-dimethyl-tetrahydro-pyran-4-ylmethyl)-4-fluoro-piperidin-4-ylmethyl]-benzamide) [39], ALA inhibited T-channels only partially (up to 40% maximal current inhibition). However, we demonstrated in current-clamp recordings that even partial T-channel inhibition by ALA effectively decreased excitability of nociceptive DRG neurons [25].

Based on our results presented herein, we propose that ALA may represent a safer class of analgesics with desirable analgesic properties by targeting T-channels in the pain pathway in the postoperative period. Although we have used T-channel blockers like TTA-P2 for the proof-of-principle in our preclinical studies of postsurgical pain [20], we predict that the use of ALA in perioperative period in humans may have fewer side effects than targeting T-channels with specific inhibitors, given that mechanism of channel block is different (partial vs. complete, respectively). Since in the present study we injected ALA systemically, it is possible that the observed effects may be a result of its action on peripheral and/or central pain pathways. This notion is based on the fact that T-channels, and particularly the Ca_V_3.2 isoform in both peripheral and central nociceptive neurons are strongly implicated in pain modulation [40]. Consistent with this idea, we have demonstrated recently that both intrathecal and local intraplantar injections of specific T-channel inhibitors effectively attenuated hyperalgesia after surgical skin incision in rats [20,26]. Toward this end, other studies have shown that systemic (intravenous) application of ALA significantly reduced firing frequency of wide dynamic range spinal trigeminal nucleus caudalis neurons in response to nociceptive and non-nociceptive mechanical stimulation in vivo suggesting that ALA suppresses trigeminal sensory transmission, possibly by blocking T-channels [41]. However, we cannot exclude the possibility that ALA may affect other ion channels in pain pathway that may work in concert with T-channels to influence neuronal excitability.

### Spontaneous pain after surgery in rats can be alleviated by systemic ALA treatment

Previous studies have shown that ALA may attenuate different types of evoked pain states in animal models. ALA has been shown to alleviate visceral pain due to colon distension in diabetic rats [9], at least partly by reducing sodium currents and downregulating expression of Na_V_1.7 and NaV1.8 channels in colonic sensory afferents of dorsal root ganglia [8] of diabetic rats. In the paclitaxel-induced neuropathy model ALA reduced pain possibly through the Nrf2 signaling pathway [10]. In addition to the measure of evoked pain described above, we also determined the effects of paw incision and ALA on nonevoked (spontaneous) pain parameters in order to better reflect the experience of human pain. Indeed, prevailing literature strongly suggests that spontaneous pain or pain at rest represents a great problem in patients after surgery, since it is notoriously resistant to the conventional treatments [42].

Unfortunately, very little is known about the molecular mechanisms and methods of measurements of spontaneous pain in animal models. Determining non-evoked pain in rodents after surgery can be performed by using conventional tests such as guarding pain test [43]. This test is performed by observing and scoring guarding behavior of incised paw in rats over a period of time, which can be time consuming and sometimes difficult to provide proper assessment of the guarding intensity. On the other hand, the dynamic weight-bearing apparatus provides automated measurement of guarding behavior after paw incision in rodents, based on paw pressures exerted against the surface of the enclosure while animals are freely moving. In this way, the assessment is unbiased and is based on data collected in 5 minute intervals. In our experiments we identified that the weight-bearing differences between unincised and incised paws in animals that received ALA were significantly lower than in animals treated with vehicle, indicating that preemptive repeated treatment with ALA effectively reduced spontaneous pain in rats after surgery.

The exact underlying mechanism for the development of spontaneous pain after surgical incision is not yet fully understood. It was previously shown that spinal sensitization occurring after plantar surgical incision is induced by repetitive firing of peripheral sensory nerves, and is maintained via AMPA subtype of glutamate receptors [44–46], leading to development of non-evoked pain and hyperalgesia [45]. We have recently discovered that Ca_V_3.2 channels contribute to the development of hyperexcitability of peripheral sensory neurons after surgical incision [20], and are important for the development and maintenance of mechanical hyperalgesia after surgery. Furthermore, presynaptic release of excitatory synaptic transmission mediated by glutamate in the dorsal horn of spinal cord is regulated by Ca_V_3.2 channels [47]. Therefore, spontaneous pain induced by spinal sensitization after surgical incision could develop, at least in part, due to Ca_V_3.2- dependent hyperexcitability of peripheral nerves [20] and/or enhanced synaptic release of glutamate in dorsal horn lamina I–II [47]. Nevertheless, additional studies are necessary to further elucidate the role of T-channels in nonevoked pain after surgery.

## Conclusion

In conclusion, our present results demonstrate that ALA reduces both evoked and spontaneous pain in a rodent postsurgical pain model. We propose that our preclinical study may lead to further investigation of analgesic properties and mechanisms of analgesic action of ALA in patients undergoing surgery. It is hoped that the use of dietary supplements like ALA may provide adequate perioperative analgesia and diminish the risk of hypersensitivity, drug addiction and tolerance resulting from opioid overuse.
